# Daylight Photodynamic Therapy: An Update

**DOI:** 10.3390/molecules25215195

**Published:** 2020-11-08

**Authors:** Chaw-Ning Lee, Rosie Hsu, Hsuan Chen, Tak-Wah Wong

**Affiliations:** 1 Department of Dermatology, National Cheng Kung University Hospital, College of Medicine, National Cheng Kung University, Tainan 704, Taiwan; joyce060324@gmail.com (C.-N.L.); hrposie@gmail.com (R.H.); hoopdebby@hotmail.com (H.C.); 2 Institute of Clinical Pharmacy and Pharmaceutical Sciences, College of Medicine, National Cheng-Kung University, Tainan 704, Taiwan; 3 Department of Biochemistry and Molecular Biology, College of Medicine, National Cheng Kung University, Tainan 701, Taiwan; 4 Center of Applied Nanomedicine, National Cheng Kung University, Tainan 701, Taiwan

**Keywords:** actinic keratosis, aminolevulinic acid, daylight, photodynamic therapy, history, nonmelanoma skin cancer

## Abstract

Daylight photodynamic therapy (dPDT) uses sunlight as a light source to treat superficial skin cancer. Using sunlight as a therapeutic device has been present for centuries, forming the basis of photodynamic therapy in the 20th century. Compared to conventional PDT, dPDT can be a less painful, more convenient and an effective alternative. The first clinical uses of dPDT on skin cancers began in Copenhagen in 2008. Currently, aminolevulinic acid-mediated dPDT has been approved to treat actinic keratosis patients in Europe. In this review article, we introduce the history and mechanism of dPDT and focus on the pros and cons of dPDT in treating superficial skin cancers. The future applications of dPDT on other skin diseases are expected to expand as conventional PDT evolves.

## 1. Introduction

**Figure 1:**
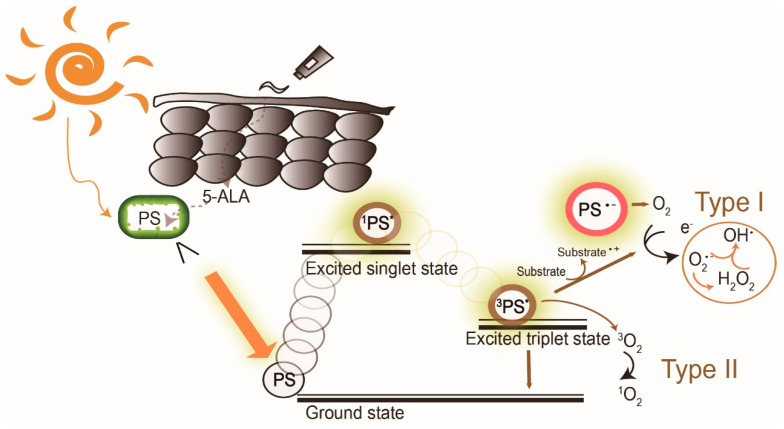
The mechanism of daylight photodynamic therapy. After daylight absorption, the photosensitizer (PS, 5-aminolevulinic acid (5-ALA), a prodrug of the real PS protoporphyrin IX, is exemplified here) is excited to a singlet state and undergoes intersystem crossing to the excited triplet-state. The triplet excited PS can react in two ways: a Type I reaction which involves the generation of superoxide anion radical (O_2_^•−^), hydrogen peroxide (H_2_O_2_), and hydroxyl radical (OH^•^) by electron transfer to molecular oxygen, and/or by the type II reaction through energy transfer to generate singlet oxygen (^1^O2). PS: photosensitizer; ^1^PS*: excited singlet state, ^3^PS* excited triplet state.

**Figure 2:**
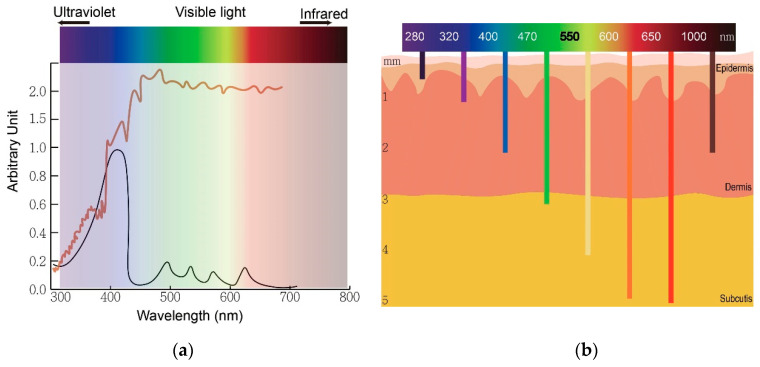
The wavelength of light determines an optimal therapeutic window of photodynamic therapy. (**a**) The absorption peaks of protoporphyrin IX (black) and sunlight spectrum (brown). (**b**) The relationship between wavelengths of light and skin penetration depth.

Photodynamic therapy (PDT) is a medical treatment utilizing photosensitizers in conjunction with a specific light source to exert cytotoxic activity in tumor cells [1]. PDT has been approved to treat superficial nonmelanoma skin cancer worldwide, to date, due to its noninvasive procedure, enhanced tumor selectivity, good to excellent cosmetic outcome and large treatment field [2,3]. Photosensitizers (PS), visible light and oxygen are the three key elements in PDT, and the combination of the three results in tumor necrosis and apoptosis. PS is excited by specific wavelengths that contain the absorption peaks of a PS in visible light (usually red or blue light), near-infrared light and even sunlight. After illumination, the PS is excited from the ground state to the triplet state (Figure 1). The excited photosensitizer (PS*) undergoes two kinds of reactions. In type I reaction, the excited PS reacts with biomolecules such as lipids, proteins and amino acids to yield the superoxide anion radical (O_2_^•−^) and HO_2_^•^, through electron transfer. O_2_^•−^ undergoes dismutation to form hydrogen peroxide (H_2_O_2_), the precursor of the highly reactive hydroxyl radical (OH^•^). OH^•^ is extremely chemically reactive to almost all biological molecules, which can achieve a better antihypoxia outcome [4]. In the type II reaction, the excited PS yields singlet oxygen (^1^O_2_) through direct energy transfer to molecular oxygen. Singlet oxygen, like the hydroxyl radical, is highly reactive. The two reactions may occur simultaneously, and the ratio of the reactions depends on the type of PS used and concentrations of substrate and oxygen. Nonetheless, the type II reaction is the principal mechanism of O_2_-dependent PDT (Figure 1) [5,6,7]. PDT with PS, especially 5-aminolaevulinic acid (5-ALA) or its ester form, methyl aminolevulinate (MAL), is widely applied to dermatologic diseases including superficial nonmelanoma skin cancers, infections, infestation diseases, inflammatory diseases and photorejuvenation. The major limitation of PDT in treating skin diseases, either benign or malignant, is the penetration depth of light. Figure 2 shows the relationship between wavelengths of light and skin penetration. As a result, PDT is approved to treat actinic keratosis (AK) and nonmelanoma skin cancers including Bowen’s disease (squamous cell carcinoma in situ), superficial basal cell carcinomas and certain thin basal cell carcinomas. Other emerging indications include extramammary Paget’s disease, cutaneous Leishmaniasis, verruca, acne vulgaris, scleroderma and vulvar lichen sclerosis et atrophicus [3].

Actinic keratosis (AK) is a common precancerous skin lesion caused by cell damage under chronic exposure to ultraviolet (UV) light from sunlight or indoor tanning. People with fair skin color (Fitzpatrick skin types I and II) are more susceptible to AK. In Australia, the prevalence of AK was estimated to be 40–50% of the population 40 years and older [8]. The dysplastic keratinocytes in AK are confined to the basal layer of the epidermis in the skin, usually less than 300 μm deep. This depth is easily reached by light with wavelengths longer than 400 nm (blue) and renders AK the first skin disease approved for PDT treatment globally. Clinically, AK lesions are dry, scaly and erythematous spots or patches located on sun-damaged skin. The AK lesions are often more easily felt than seen. They are rough and dry to touch. PDT allows treatment of large and multiple lesions. The treatment outcome is not inferior to conventional therapies such as surgery and cryotherapy while the cosmetic outcome is superior to these conventional treatments.

In conventional PDT (cPDT), after the application of prodrug ALA for 3 h, the metabolic product protoporphyrin (PpIX) accumulates in the mitochondria of a cell. PpIX is photoactivated by irradiation with specific wavelengths of light. The light source must correspond with the absorption peaks of PpIX. PpIX has absorption peaks at 405, 510, 545, 580, 635 nm (Figure 2a). The Soret band of PpIX is 405 nm but a 635 nm light source is widely used in clinics for the purpose of a deeper light penetration. Blue (410 nm) and red (635 nm) light from lasers, lamps and LEDs are most commonly used. After irradiation, reactive oxygen species (ROS) by type I reaction and singlet oxygen by type II reaction cause cell death (Figure 1). The therapeutic depth from the skin is 1~2 mm with blue light and 1~6 mm with red light, limiting PDT usage in more superficial cutaneous lesions (Figure 2b). The light sources are not widely available in every clinic. cPDT is time-consuming (usually 3 h occlusion of ALA), staff skills dependent and relatively limited in the treatment area under a fixed light source. Although cPDT is a convenient field-directed therapy with a good cosmetic outcome, the discomfort and pain during and after cPDT impedes its usage. Daylight emits UV, visible and infrared lights which cover the absorption peaks of most, if not all, available photosensitizers nowadays (Figure 2b). Patients experienced less pain with daylight PDT (dPDT) and were more satisfied with the procedure. A 2-h daylight exposure is sufficient for treatment and weather does not have a large impact on dPDT in most countries. This review updates the current and future role of dPDT in nonmelanoma skin cancers, especially actinic keratosis and other skin diseases.

## 2. History of PDT

The earliest known reports of phototherapy, previously known as heliotherapy, were in ancient Greece, Egypt and India in the treatment of various diseases such as vitiligo, psoriasis and skin cancer [9]. Hippocrates, the Father of Medicine, spread the teachings of heliotherapy, using the sun as a therapeutic agent. Phototherapy was popularized as a medical treatment by the Danish physician Niels Finsen who received the Nobel prize in 1903 for his work in carbon arc phototherapy for skin tuberculosis [10]. Incidental findings of toxicity on paramecium via fluorescence, a product of light and acridine together, were observed in an experiment led by Professor Herman von Tappeiner and his student, Oscar Raab. In 1904, Von Tappeiner and Joldbaur reported that oxygen was required for photosensitization. In 1907, Von Tappeiner first coined the term photodynamic therapy in his book narrating experiments of oxygen-dependent photosensitization on skin cancer, lupus of the skin and condyloma of the female genitalia [11].

Hematoporphyrin (hp) was first produced by Scherer in 1841 [12]. In 1908, Hausmann studied the phototoxic effects on white mice and found that the effects were dependent on the doses of photosensitizer and light. The first proof of porphyrins as photosensitizers applied on humans was in 1913 when Friedrich Meyer-Betz injected himself with 200 mg hematoporphyrin and experienced severe photosensitive skin reactions for two months [13]. In 1924, Policard first observed that UV radiation produced red fluorescence in experimental rat sarcomas and correctly attributed the finding to the accumulation of hp. In 1942, Auler and Banzer were the first to observe PDT with hp on tumors. An evolutionary finding was made by Schwartz, in 1995, who isolated hp derivatives (HpD) which were more enhanced in tumor-localizing properties and phototoxicity than hp itself. The selective and destructive effect of HpD on breast cancer and bladder tumors was investigated by Lipson et al. [14] and Kelly and Snell [15] in 1966 and 1976, respectively. The modern era of PDT began with the first systematic human trials for PDT on cutaneous and subcutaneous cancer performed by Dougherty et al. in 1978 [16]. Dougherty and his coworkers also purified HpD and produced Photofrin, which was the first clinically approved photosensitizer for the treatment of cancer.

cPDT with Photofrin was first approved by the US Food and Drug Administration (FDA) in 1995 [17]. Photofrin (Porfimer sodium; Axcan Pharma, Inc., Ontario, CA, USA) is injected intravenously and irradiated with 630 nm wavelength laser light [18]. However, increased skin photosensitivity persists over two months after the administration of Photofrin. Therefore, second-generation photosensitizers were developed over the decades. Currently, three ALA based photosensitizers are approved to treat skin cancers with PDT in the United States, including 5-ALA (Levulan^®^, DUSA Pharma Inc., New York, NY, USA), methyl-5 aminolevulinic acid (MAL) (Metvix^®^, Photocure ASA, Oslo, Norway) and a new nanoemulsion 10% ALA hydrogel (BF-200, Ameluz^®^, Biofrontera Bioscience GmbH, Overland Park, KS, USA). The second-generation photosensitizers are more effective and less harmful than Photofrin.

**Figure 3:**
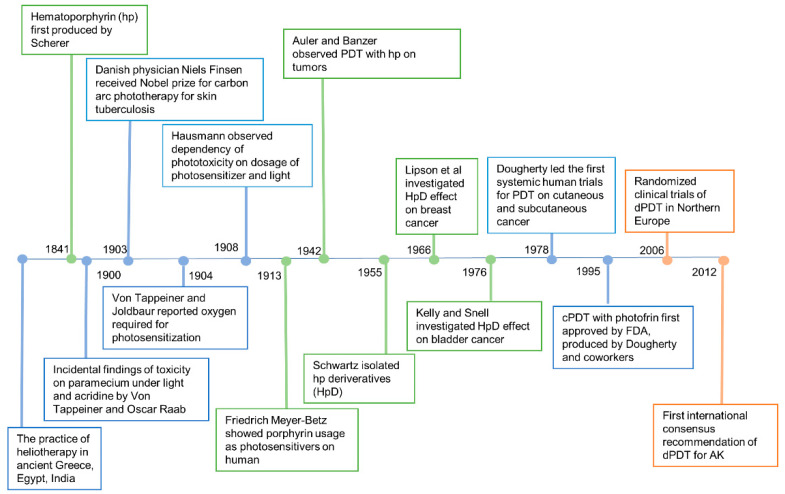
History of photodynamic therapy.

The original form of ALA in topical creams, ointments or gels is very unstable and needs to be used within hours to days after production due to its chemical properties [19]. ALA undergoes dimerization irreversibly to form 2,5-dicarboxyethyl-3,6-dihydropyrazine, which spontaneously oxidizes to 2,5-dicarboxyethylpyrazine in aqueous solutions. In addition, ALA is a zwitterion at physiological pH which impairs its penetration through the lipophilic stratum corneum [20]. From a pharmaceutical perspective, epidermal penetration and stability of the prodrug and its vehicle determine the efficacy of ALA-PDT [21]. By adding methyl esters, MAL became one of the more lipophilic ALA derivatives with better stability and deeper penetration than ALA. However, the ester is cleaved away before MAL can enter the heme biosynthetic pathway. As a result, less PpIX is formed with MAL as compared to ALA after the same incubation time [19]. A new 10% ALA hydrogel (BF-200) was developed [19]. The oil-in-water nanoemulsion hydrogel contains 7.8% of free ALA that connects to the polar phosphatidylcholine head groups of lipids and extends the ALA stability from several days to over two years [19]. The nanoemulsion BF-200 is approximately 20 nm, contains a monolayer of phosphatidylcholine molecules and a long chain of MiglyolR^®^812 with many caprylic acid (C8) and capric acid (C10) and fatty acid (FA) molecules. BF-200 nanovesicles rapidly fuse with stratum corneum (SC) lipid layers and the shorter FA integrates into the SC, increasing SC fluidity and ALA penetration through the epidermis [19]. Figure 3 demonstrates the evolution of ALA based photosensitizers. dPDT with MAL cream (Metvix^®^, TM, Photocure ASA, Oslo, Norway) and BF-200 ALA (Ameluz^®^ Biofrontera, Leverkusen, Germany) are licensed to treat AK in Europe [22]. Dirschka et al. revealed a noninferiority of BF-200 ALA to MAL with dPDT in the treatment of mild-to-moderate AK [19,23]. At a three-month follow-up, 79.8 and 76.5% of the AK lesions treated with BF-200 ALA gel-dPDT and with MAL-dPDT showed complete remission, respectively. The BF-200 ALA gel group had lower recurrence rates after a one-year follow-up. A new self-adhesive 5-ALA patch form (Alacare^®^; Photonamic, Pinneberg, Germany) is approved to treat AK and is advantageous since pretreatment is unnecessary [3].

cPDT uses artificial light to activate the photosensitizers. However, due to the significant pain with cPDT, sunlight was considered an alternative light source. Weigel et al. led the pioneer work in northern Europe demonstrating the efficacy, safety and benefits of dPDT [24]. An international consensus recommendation of dPDT for actinic keratosis, penned by The International Society for Photodynamic Therapy in Dermatology, was published in 2012 [25]. Since then, consensus recommendations in Australia, UK, Spain and other countries have been made according to the geological and climatic changes in individual countries.

## 3. Selectivity of PDT on Cancer Cells

**Figure 4:**
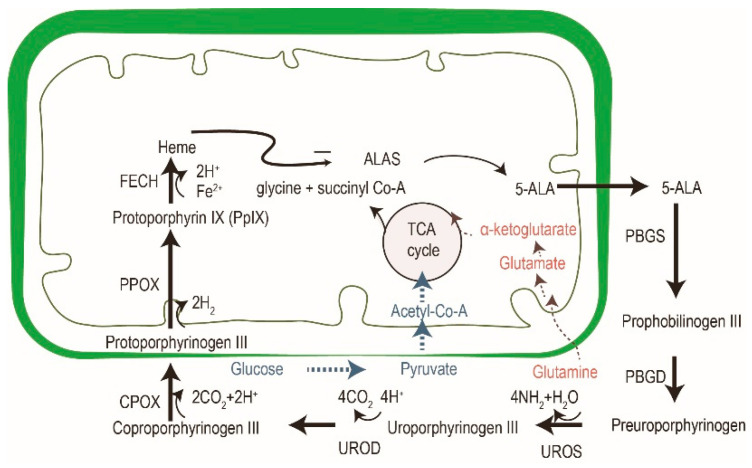
The cross-talk of glucose, glutamine and porphyrin heme biosynthesis pathways. In addition to porphyrin synthesis via the tricarboxylic cycle acid (TCA) cycle, the accumulation of PpIX in tumor cells might be explained by an increase of glycolysis (in blue) and glutaminolysis (in red). Modified from reference 24.

5-aminolevulinic acid (ALA) is the most common photosensitizer used in the dermatologic field nowadays. It is a prodrug that converts into protoporphyrin IX (PpIX) via an intrinsic cellular heme biosynthetic pathway (Figure 4). PpIX receives feedback inhibition by the intracellular level of heme. More exogenous PpIX accumulates in the inflammatory, premalignant and malignant cells than in normal epidermal cells due to the alterations in enzyme activity of heme biosynthesis. The limited capacity of ferrochelatase (FECH) and increased enzymatic activity of ALA dehydratase (ALAD), porphobilinogen deaminase (PBGD) and uroporphyrinogen decarboxylase (UROD) result in the accumulation of exogenous PpIX in tumor cells [26,27]. Another possible explanation for the increased accumulation in neoplastic cells could be altered ALA or porphyrin uptake or decreased PpIX efflux mechanisms. Recent studies have also suggested that enhanced glycolysis in tumor cells, and the subsequent switching from glucose tricarboxylic cycle acid (TCA) to aerobic glycolysis, may activate the heme biosynthesis pathway to remove the TCA metabolites [25]. The increased activation of the heme biosynthesis pathway may enhance PpIX accumulation due to limited FECH.

## 4. Advantages of dPDT

The major drawback of cPDT is the intolerable burning or stinging pain during or after treatment. Pain during irradiation was thought to be related to a large accumulation of PpIX after a long incubation time, especially on the acral and genital skin where nerve fibers are abundant [28,29,30]. The mechanism that causes pain with PDT is not well understood. Reactive oxygen species (ROS) were believed to be the main contributor in PDT-induced pain by increasing phosphorylation of *N*-methyl-d- involved in the pathway of nociception [31,32,33]. ROS-mediated tissue damage also results in the aspartate (NMDA), transient receptor potential vanilloid type 1 (TRPV1) and transient receptor potential ankyrin 1 (TRPA1) receptors release of proinflammatory cytokines including interleukin 1, 6 and tumor necrosis factor-alpha [31]. Both effects of ROS contribute to the stimulation of nociceptors. Others have postulated that local hypoxia, after depletion of oxygen in the process of PpIX photoactivation and tumor destruction, can decrease pH in tissue and trigger pain signals [33,34]. Many studies revealed more severe pain with ALA compared to MAL [35], perhaps since ALA is actively transported into the peripheral nerve endings, probably to the unmyelinated afferent c-fibers, and induces nerve stimulation during irradiation [26]. Whether pain increases with light fluences (J/cm^2^) or not is controversial [35]. Wang et al. theorized that the pain with PDT positively correlates with fluence rate (W/m^2^ or mW/cm^2^) and dose below a certain threshold (around 60 mW/cm^2^, 50 J/cm^2^) and reaches a plateau beyond the threshold, possibly due to the desensitization of nociceptors or limited cell capacity to produce ROS. Grapengiesser et al. found that patients who received cPDT on actinic keratosis (AK) lesions suffered more pain (higher visual analog scale (VAS)) than those treated on Bowen’s disease and basal cell carcinoma (BCC) lesions [36]. Studies proposed that cPDT on well-innervated locations, such as the face, hands and perineal regions, were more painful [36]. Wiegell et al. proposed that continuous PpIX activation without occlusion and small accumulation of PpIX makes dPDT a less painful, but as effective, alternative to cPDT [24].

dPDT uses daylight as an alternative light source for PDT in the treatment of actinic keratosis (AK) and other skin lesions. dPDT emerged in Europe around 2006 [24]. In contrast with the three-hour incubation in cPDT, dPDPT only requires a 30-min incubation. The reason for the shortened incubation is the constant low-level activation of PpIX during daylight exposure. In cPDT, approximately 80% of PpIX is activated simultaneously within a few minutes to an illumination dose of 10 J/cm^2^ [37]. In dPDT, however, the continuous activation of PpIX matches the speed of PpIX formation. No PpIX is accumulated in the skin, contributing to the reduced pain during the treatment. The visible light (wavelength: 380 to 700 nm) is used in dPDT. The wavelength is different from UV light (100 to 380 nm,) thus sunscreen is recommended to block broadband UV to prevent UV damage under daylight exposure. dPDT is also more convenient as it can be performed by the patients themselves at home. An investigation conducted in the UK reported that patients were willing to perform dPDT at home with 82% being happy or very happy with the service due to minimal adverse events, good efficacy and cosmetic outcome [38]. Rubel et al. also revealed less scarring or hypopigmentation of dPDT compared to cPDT [39]. Not only is dPDT less painful and larger in the treatment area than cPDT but the incubation time is shorter than cPDT (Table 1).

## 5. Clinical Applications of dPDT

### 5.1. Actinic Keratosis

**Figure 5:**
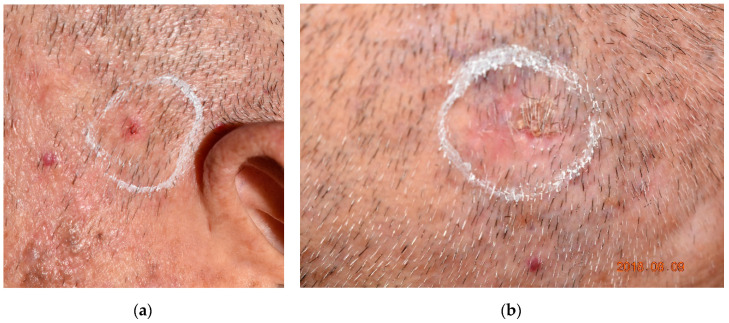
Clinical pictures of actinic keratosis (AK). (**a**) An erythematous AK locates on sun-damaged skin in an elderly patient. (**b**) A thick keratotic AK on the scalp of the same patient. In a thick AK, the hyperkeratotic skin surface prevents sufficient transdermal delivery of a photosensitizer for photodynamic therapy. The rough surface is usually removed by curettage before the application of a photosensitizer. AK lesions are highlighted by circles.

AK is a precancerous lesion (Figure 5) that occurs on sun-damaged skin. AK lesions are prone to field cancerization and around 0.5–10% may develop into squamous cell carcinoma (SCC) [40,41,42]. Field cancerization was first named by Slaughter in 1953 when studying the epithelium of oral SCC. Slaughter implied that oral SCC arose from multifocal areas of precancerous changes under carcinogenic exposure rather than from key malignant cells. Braakhuis et al. defined field cancerization as a preneoplastic status. Therefore, it does not have the ability of invasion or metastasis [43]. However, the field may or may not have visible histopathological changes of dysplasia and some are found to have genetic alterations. Molecular studies raised the issue of genetic mutations in the malignant transformation of normal cells [41]. The mutation in the gene TP-53 is well documented and presents more in photodamaged skin and head and neck cancer [41]. Studies also proposed that it takes more than one genetic alteration to turn normal tissue into subclinical lesions then into clinical or even invasive cancer. These genes include the famous TP-53 and the p-16, antiapoptotic complex Bcl-2 and cyclin D1 genes [41]. The field originates from a stem cell that acquires one or more genetic alterations. Sub-clones may originate in the field after the acquisition of new gene alterations and may undergo carcinomatous transformation [41]. Thus, field-directed therapy is a means to treat not only clinically visible lesions but also nonvisible subclinical lesions within the field. If a new AK lesion is developed followed by another AK in the same chronically photodamaged skin area, the risk of cancerous change of the third or fourth AK presented in this field is high. Field-directed therapy in photodamaged areas in AK patients [44] has been widely used in recent years. Imiquimod, Ingenol mebutate, PDT and cryotherapy are considered field-directed therapies.

The Olsen grading system grades AK into three grades: grade 1, slightly palpable; grade 2, moderately thick; and grade 3, very hyperkeratotic lesions [45]. dPDT on AK is supported by evidence from randomized studies and established consensus from the Western countries with impressive cosmetic results and few side effects [28,42]. dPDT is especially suitable for patients with multiple lesions on large actinic damaged skin, recommended by the International Society for Photodynamic Therapy in Dermatology (ISPTD) [28]. Multiple randomized trials revealed no statistical difference in treatment response between cPDT and dPDT. The complete remission rate ranged from 70% to 92% [24,39,46]. However, it was recommended that dPDT should be applied to superficial to moderate thick AK (grade 1–2 AK) instead of thick AK (grade 3 AK) [42,47]. The subgroup analysis of dPDT-treated AK revealed higher response rates of thin AK (76%) but lower response rates of moderate to thick AK, similar to the results of cPDT [42,47]. Perez et al. reported similar efficacy of dPDT and cPDT for grades 1 and 2 AK but revealed inferiority of dPDT to cPDT for grade 3 AK [48]. Notably, Fai et al. also reported poor response of dPDT on squamous cell carcinoma (SCC) [49]. Thus, the efficacy of treatment on grade 3 AK is still controversial. The randomized studies on dPDT for treatment of AK were conducted in Europe, Australia, Scotland, America and China [28,42,46,50,51,52,53].

#### 5.1.1. Treatment Guidelines for Actinic Keratosis

The consensus recommendations for treating AK with dPDT in Europe, Australia and Canada are similar [28,46]. Many studies are dedicated to improving the efficacy and safety of dPDT. The current protocols are summarized in Table 2.

#### 5.1.2. Timing

The Australian consensus suggested that dPDT can be performed all year under any weather except for rainy days [42]. dPDT can be performed almost all year round in countries south of latitude 45° N but not in northern countries during the winter [54]. dPDT usually takes place outdoors but can also be performed in a greenhouse where the glass absorbs most of the UV [54].

#### 5.1.3. Skin Preparation

Different methods have been adopted to improve skin penetration of ALA. Curettage before applying ALA is the most common method used for skin preparation, especially for hyperkeratotic thick AK. Microneedling the skin can also help in ALA transdermal drug delivery. Torezan et al. conducted a pilot split-face study on 10 patients with actinically damaged skin to compare mild curettage before applying MAL-ALA on one side of the face with microneedling, with 1.5-mm microneedles on the other half after MAL application [55]. Microneedling-assisted PDT side showed superior cosmetic results to curettage-assisted MAL-PDT. However, one patient developed a skin infection on the side with microneedling. Erythema, edema, crusting and pain were noted more often with microneedling than with curettage [55,56]. In a randomized clinical trial of 25 patients with around 370 grade 1 AKs and 30 grade 2 to 3 AKs, Heerfordt et al. [56] compared dPDT with curettage before applying MAL for 30 min to MAL for one hour without prior curettage on two even-sized areas on the face and scalp of the same patients. Both areas received two hours of daylight exposure. The results showed no difference in cure rate and adverse effects three months after dPDT. Pretreatment with calcipotriol could enhance the efficacy of dPDT but could also increase the intensity and risk of erythema and desquamation [57]. Nissen et al. reported that one week of pretreatment with topical 5% 5-fluorouracil (5-FU) twice daily before dPDT had significantly higher response rates than dPDT alone at three-month follow-up. 5-FU could be applied to lesions on limbs where dPDT is usually less responsive [52].

#### 5.1.4. Incubation Time for Photosensitizing Agents

Sunlight exposure should be initiated no longer than 30 min after applying MAL cream to avoid a higher risk of increased pain [46]. Increased pain is supposed to result from a greater accumulation of PpIX before sun exposure [55]. However, a longer incubation time may increase MAL absorption in AK without curettage [56].

#### 5.1.5. Sunlight Exposure

To generate sufficient PpIX in AK, the European consensus recommended two hours of daylight exposure without interruption [45]. However, sunlight exposure longer than two hours is not recommended due to a higher risk of sunburn. The photosensitizing agents are then washed completely. The patients are educated to stay indoors and to apply sunscreen to avoid further sun damage. O’Mahoney et al. reported dosimetry methods measuring the light exposure in dPDT. The two devices used to measure spectral irradiance were the scanning spectroradiometer and the array spectrometer [22,39]. However, spectrometers are not readily available and can be replaced by handheld broadband radiometers and luxmeters [22]. The threshold for an effective dPDT dose was estimated to be 8 J/cm^2^ for treatment applied at the irradiance of 130 W/m^2^ [22]. Wiegell et al. reviewed dosimetry in dPDT and cited that the minimal threshold PpIX-effective light dose was 3.5 J cm^−2^ when monitored with a wristwatch device [22,47]. Notably, measurements on the wrist should be considered as only 50% of the light dose measured on the scalp [22].

#### 5.1.6. Treatment Sessions/Follow-Up

The number of treatment sessions and the intervals between treatments vary in different studies [46]. Some studies performed one-time dPDT on grade 1 AKs, and two times on grade 2 and 3 AKs [46,58]. An intrapatient randomized trial involving 31 patients showed no superiority of two-treatment sessions to single sessions of MAL dPDT for AKs of the face and scalp [59]. The intervals between treatments varied from one day to one month [46]. Patients were followed up in clinics for at least half a year in case of residual or recurrent AK. Long-term follow-up for 12 months revealed similar efficacy and recurrence rates in AK on the face and the scalp with dPDT and cPDT [50].

Bernad et al. studied whether repeated treatments of field cancerization with dPDT were effective in preventing new AK and keratinous carcinoma in 24 patients received organ transplants [60]. The randomized, intrasubject controlled, evaluator-blind, split-face and/or scalp dPDT was compared to the lesion-directed cryotherapy control side. The dPDT treated side showed lower means of new lesions compared to the control side at three months, nine months and 15 months, and nonsignificance at 21 months. The results suggested the potential of repeated dPDT to prevent new AK and keratinous carcinoma in high-risk patients.

### 5.2. Actinic Cheilitis

Actinic cheilitis (AC) is a precancerous disease on the vermilion border caused by long term sunlight exposure, with a predilection of lower lip involvement. The risk of progression to lip SCC has been reported [61]. Some case reports showed success in treating AC with cPDT [62,63]. However, local anesthesia is usually needed for cPDT because the lip is full of nerve endings. Levi et al. reported complete remission of AC in 10 of 11 patients after a median of two sessions of dPDT. After applying MAL cream, patients were exposed to sunlight in the garden from 8 to 11 a.m and underwent one to six sessions. Patients treated themselves at home after the first session. Only mild erythema, pain and edema were observed during and after the procedure [61].

### 5.3. Xeroderma Pigmentosum (XP)

XP is a genetic disorder with a DNA repair defect characterized by extreme sensitivity to ultraviolet (UV) light. The extreme sensitivity to UV light results in multiple skin cancers, such as AK, squamous cell carcinoma or melanoma at a young age. A publication in Africa showed promising results of dPDT for AK in XP patients [64]. A total of 13 XP children were applied with either ALA or MAL followed by two hours of indoor light exposure. Weekly imiquimod was not discontinued prior to and after PDT. Crusting and scaling at the treated area were seen for two days. Improvement was seen after two weeks and no adverse events were seen at three-months follow-up [64]. Alicia et al. studied the efficacy of cPDT on dermal fibroblasts from XP [65]. Cancer-associated fibroblasts (CAF) and normal fibroblasts were immersed in MAL (1mM) for five hours and then exposed to different light doses (from 1.8 to 16.7 J/cm^2^) with a 384 light-emitting diode (LED) matrix emitting 636 nm ± 17 nm red light. Results showed a significant difference in the decrease of survival between CAF and normal fibroblasts at 40 and 5%, respectively. The authors concluded that the selective cytotoxicity might be related to the decrease of inactive caspase-3 followed by increased expression of cleaved Poly ADP Ribose Polymerase (PARP) after cPDT in CAF.

### 5.4. Bowen’s Disease

**Figure 6:**
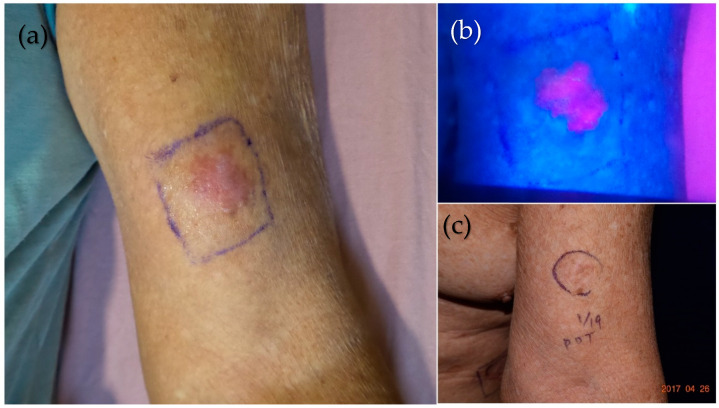
dPDT treatment on an elderly chronic arsenism patient with multiple Bowen’s diseases (BDs). (**a**) One BD on the left upper arm was marked to show the drug application area. (**b**) To improve the transdermal delivery of the photosensitizer, the lesion was occluded for one hour after being slightly curetted. A vivid pink fluorescence of PpIX was well localized within the tumor under Wood’s lamp examination. (**c**) The lesion was clinically clear after one treatment of 2 h of exposure to sunlight. No recurrence was noted one year after dPDT.

The European consensus also recommended treating Bowen’s disease (BD, or squamous cell carcinoma in situ) with cPDT and dPDT [3,66]. Roba et al. reported the first BD patient treated with MAL-dPDT under two hours of sunlight exposure. The resolution of BD was achieved at a three-month follow-up [67]. A study in Brazil [68] enrolled 24 BD lesions which were treated with two sessions of MAL-dPDT with a one-week interval. 25% of BD showed complete remission and 33% showed >75% partial response. Lesions located at sun-exposed areas, such as the upper limbs and head/neck area, showed better response than those on the trunk and lower limbs. Adverse events were minimal and tolerable. We treated patients successfully who suffered from chronic arsenism with multiple BDs with dPDT (Figure 6, unpublished data).

### 5.5. Basal Cell Carcinoma

Wiegell et al. in Denmark treated 32 superficial or small nodular basal cell carcinoma (BCC) with two sessions of MAL-dPDT with a one-week interval and achieved 74% complete response (CR) at 12-month follow-up [69]. The skin was exposed to 2.5 h of sunlight with a mean effective light dose of 10.8 J/cm^2^. No pain was reported in 72% of the treatments. The pain was mild (0–4/10 visual analog scale) if reported. All patients reported a good or excellent cosmetic outcome. MAL-dPDT combined with imiquimod 5% for superficial BCC was reported to be superior to dPDT alone at 12-month follow-up (CR response of 91.3% versus 83.4%) [70]. dPDT was not recommended for nodular BCC. Fai et al. reported no response after dPDT on the nodular BCC formed in the treatment field of AK [46,49].

### 5.6. Benign Skin Diseases

dPDT has been applied to benign skin lesions such as acne vulgaris and photorejuvenation. A randomized clinical study, conducted in China, compared cPDT and dPDT for acne vulgaris and revealed no statistical difference in objective treatment response rate between the dPDT group and cPDT group at weeks two, four and six [71] with a two-week treatment interval. As for photorejuvenation, an extensive review performed by Le Pillouer-Prost et al. showed a high level of efficacy of cPDT and dPDT to improve skin tone, lentigo, skin roughness, texture, and fine wrinkles because of the effects of dermal remodeling [72]. Philipp-Dormston et al. also reported that in addition to clearance and prevention of AK, skin rejuvenation was demonstrated in most studies after dDPT [73].

## 6. Conclusions

dPDT using natural sunlight to activate photosensitizers is advantageous in its lower cost and reduced pain. It is a promising method to treat AK under the concept of field cancerization. Current studies suggest that the prognosis of dPDT is noninferior to cPDT. dPDT is licensed to treat AK in Europe with the support of randomized trials and consensus guidelines. There are limited reports on PDT on other skin tumors or inflammatory skin diseases other than AK. Based on the history of the development of cPDT, we can expect that the application of dPDT will grow exponentially shortly. Further noninferiority studies between dPDT and cPDT are warranted.

## Figures and Tables

**Table 1:** Advantages and disadvantages of cPDT versus dPDT.

	dPDT	cPDT
Treatment area	Large	Small
Indication	Thin AK, superficial BCC	Thin AK, superficial BCC
Incubation time	Nonocclusive 30 min	Occlusive 3 h
Location of the treatment process	Outdoor or greenhouse	Indoor
Response rate in AK	Noninferior to cPDT	Similar to dPDT
Adverse events	Almost pain-free and less skin reactions	Moderate to severe pain

AK: actinic keratosis; BCC: basal cell carcinoma.

**Table 2:** Guidelines of dPDT on actinic keratosis from different consensus recommendations.

	Recommendations	Reference
1. Pretreatment		
Clean the treatment area		
Sunscreen	Apply chemical sunscreens (e.g., sun protection factor 30–50, broad-spectrum) to both treatment area and nontreatment sun-exposed area at least 15 min before light exposure. Patients should avoid physical sunscreens such as zinc oxide, titanium dioxide.	[20]
2. Remove scales or crusts on the treatment area	To assist the penetration of photosensitizing agents into the skin	
Skin preparation-mechanical or chemical microneedling, abrasion or curettage	Curettage is the most recommended method	[38]
3. Application of photosensitizing agents	Incubation time: without occlusion for 30 min MAL cream applied to the lesions or whole area of field cancerization”. A thin layer (0.1–0.2 mm) of MAL cream is sufficient. Expert opinion: 1 to 2 g of MAL cream applied for the whole face. We suggest applying the cream one cm beyond the lesion.	[29,38]
4. Sunlight exposure	2 h (Australia) Range from 1.5 h to 2.5 h, up to 4 h	[38,39]
5. Washing off all photosensitizer after sunlight exposure	Almost pain-free and fewer skin reactions	

## References

[1] Dougherty T.J., Gomer C.J., Henderson B.W., Jori G., Kessel D., Korbelik M., Moan J., Peng Q. (1998). Photodynamic therapy. J. Natl. Cancer Inst..

[2] Lopez R.F., Lange N., Guy R., Bentley M.V. (2004). Photodynamic therapy of skin cancer: Controlled drug delivery of 5-ALA and its esters. Adv. Drug Deliv. Rev..

[3] Morton C.A., Szeimies R.M., Basset-Seguin N., Calzavara-Pinton P., Gilaberte Y., Haedersdal M., Hofbauer G.F.L., Hunger R.E., Karrer S., Piaserico S. (2019). European Dermatology Forum guidelines on topical photodynamic therapy 2019 Part 1: Treatment delivery and established indications—Actinic keratoses, Bowen’s disease and basal cell carcinomas. J. Eur. Acad. Dermatol. Venereol..

[4] Zhuang Z., Dai J., Yu M., Li J., Shen P., Hu R., Lou X., Zhao Z., Tang B.Z. (2020). Type I photosensitizers based on phosphindole oxide for photodynamic therapy: Apoptosis and autophagy induced by endoplasmic reticulum stress. Chem. Sci..

[5] Castano A.P., Demidova T.N., Hamblin M.R. (2004). Mechanisms in photodynamic therapy: Part one-photosensitizers, photochemistry and cellular localization. Photodiagnosis Photodyn. Ther..

[6] Noodt B.B., Berg K., Stokke T., Peng Q., Nesland J.M. (1996). Apoptosis and necrosis induced with light and 5-aminolaevulinic acid-derived protoporphyrin IX. Br. J. Cancer.

[7] Ding H., Yu H., Dong Y., Tian R., Huang G., Boothman D.A., Sumer B.D., Gao J. (2011). Photoactivation switch from type II to type I reactions by electron-rich micelles for improved photodynamic therapy of cancer cells under hypoxia. J. Control. Release.

[8] Salasche S.J. (2000). Epidemiology of actinic keratoses and squamous cell carcinoma. J. Am. Acad. Dermmatol..

[9] Daniell M.D., Hill J.S. (1991). A history of photodynamic therapy. Aust. N. Z. J. Surg..

[10] Urbach F., Forbes P.D., Davies R.E., Berger D. (1976). Cutaneous photobiology: Past, present and future. J. Investig. Dermatol..

[11] Ackroyd R., Kelty C., Brown N., Reed M. (2001). The history of photodetection and photodynamic therapy. Photochem. Photobiol..

[12] Ormond A.B., Freeman H.S. (2013). Dye Sensitizers for Photodynamic Therapy. Materials.

[13] Abdel-Kader M.H., Abdel-Kader M.H. (2014). History of Photodynamic Therapy. Photodynamic Therapy: From Theory to Application.

[14] Lipson R.L., Baldes E.J., Gray M.J. (1967). Hematoporphyrin derivative for detection and management of cancer. Cancer.

[15] Dougherty T.J. (1987). Studies on the structure of porphyrins contained in Photofrin II. Photochem. Photobiol..

[16] Kessel D. (2019). Photodynamic Therapy: A Brief History. J. Clin. Med..

[17] Huang Z. (2005). A review of progress in clinical photodynamic therapy. Technol. Cancer Res. Treat..

[18] Baskaran R., Lee J., Yang S.G. (2018). Clinical development of photodynamic agents and therapeutic applications. Biomater. Res..

[19] Reinhold U. (2017). A review of BF-200 ALA for the photodynamic treatment of mild-to-moderate actinic keratosis. Future Oncol..

[20] Tewari K.M., Eggleston I.M. (2018). Chemical approaches for the enhancement of 5-aminolevulinic acid-based photodynamic therapy and photodiagnosis. Photochem. Photobiol. Sci..

[21] Tsai J.C., Chen I.H., Wong T.W., Lo Y.L. (2002). In vitro/in vivo correlations between transdermal delivery of 5-aminolaevulinic acid and cutaneous protoporphyrin IX accumulation and effect of formulation. Br. J. Dermatol..

[22] O’Mahoney P., Khazova M., Eadie E., Ibbotson S. (2019). Measuring Daylight: A Review of Dosimetry in Daylight Photodynamic Therapy. Pharmaceuticals.

[23] Dirschka T., Ekanayake-Bohlig S., Dominicus R., Aschoff R., Herrera-Ceballos E., Botella-Estrada R., Hunfeld A., Kremser M., Schmitz B., Lübbert H. (2019). A randomized, intraindividual, non-inferiority, Phase III study comparing daylight photodynamic therapy with BF-200 ALA gel and MAL cream for the treatment of actinic keratosis. J. Eur. Acad. Dermatol. Venereol..

[24] Wiegell S.R., Haedersdal M., Philipsen P.A., Eriksen P., Enk C.D., Wulf H.C. (2008). Continuous activation of PpIX by daylight is as effective as and less painful than conventional photodynamic therapy for actinic keratoses; a randomized, controlled, single-blinded study. Br. J. Dermatol..

[25] Yang X., Palasuberniam P., Kraus D., Chen B. (2015). Aminolevulinic Acid-Based Tumor Detection and Therapy: Molecular Mechanisms and Strategies for Enhancement. Int. J. Mol. Sci..

[26] Chaves Y.N., Torezan L.A., Niwa A.B., Sanches Junior J.A., Festa Neto C. (2012). Pain in photodynamic therapy: Mechanism of action and management strategies. An. Bras. Dermatol..

[27] Peng Q., Berg K., Moan J., Kongshaug M., Nesland J.M. (1997). 5-Aminolevulinic acid-based photodynamic therapy: Principles and experimental research. Photochem. Photobiol..

[28] Wiegell S.R., Wulf H.C., Szeimies R.M., Basset-Seguin N., Bissonnette R., Gerritsen M.J., Gilaberte Y., Calzavara-Pinton P., Morton C.A., Sidoroff A. (2012). Daylight photodynamic therapy for actinic keratosis: An international consensus: International Society for Photodynamic Therapy in Dermatology. J. Eur. Acad. Dermatol. Venereol..

[29] Morton C.A., Wulf H.C., Szeimies R.M., Gilaberte Y., Basset-Seguin N., Sotiriou E., Piaserico S., Hunger R.E., Baharlou S., Sidoroff A. (2015). Practical approach to the use of daylight photodynamic therapy with topical methyl aminolevulinate for actinic keratosis: A European consensus. J. Eur. Acad. Dermatol. Venereol..

[30] Wong T.W., Sheu H.M., Lee J.Y., Fletcher R.J. (2001). Photodynamic therapy for Bowen’s disease (squamous cell carcinoma in situ) of the digit. Dermatol. Surg..

[31] Wang B., Shi L., Zhang Y.F., Zhou Q., Zheng J., Szeimies R.M., Wang X.L. (2017). Gain with no pain? Pain management in dermatological photodynamic therapy. Br. J. Dermatol..

[32] Babes A., Sauer S.K., Moparthi L., Kichko T.I., Neacsu C., Namer B., Filipovic M., Zygmunt P.M., Reeh P.W., Fischer M.J. (2016). Photosensitization in Porphyrias and Photodynamic Therapy Involves TRPA1 and TRPV1. J. Neurosci..

[33] Borgia F., Giuffrida R., Caradonna E., Vaccaro M., Guarneri F., Cannavò S.P. (2018). Early and Late Onset Side Effects of Photodynamic Therapy. Biomedicines.

[34] Mikolajewska P., Iani V., Juzeniene A., Moan J. (2009). Topical aminolaevulinic acid- and aminolaevulinic acid methyl ester-based photodynamic therapy with red and violet light: Influence of wavelength on pain and erythema. Br. J. Dermatol..

[35] Ang J.M., Riaz I.B., Kamal M.U., Paragh G., Zeitouni N.C. (2017). Photodynamic therapy and pain: A systematic review. Photodiagnosis Photodyn. Ther..

[36] Grapengiesser S., Ericson M., Gudmundsson F., Larkö O., Rosén A., Wennberg A.M. (2002). Pain caused by photodynamic therapy of skin cancer. Clin. Exp. Dermatol..

[37] Wulf H.C. (2019). Daylight PDT acts by continuous activation of PpIX. Photodiagnosis Photodyn. Ther..

[38] McLellan L.J., O’Mahoney P., Logan S., Yule S., Goodman C., Lesar A., Fullerton L., Ibbotson S., Eadie E. (2019). Daylight photodynamic therapy: Patient willingness to undertake home treatment. Br. J. Dermatol..

[39] Rubel D.M., Spelman L., Murrell D.F., See J.A., Hewitt D., Foley P., Bosc C., Kerob D., Kerrouche N., Wulf H.C. (2014). Daylight photodynamic therapy with methyl aminolevulinate cream as a convenient, similarly effective, nearly painless alternative to conventional photodynamic therapy in actinic keratosis treatment: A randomized controlled trial. Br. J. Dermatol..

[40] Wenande E., Phothong W., Bay C., Karmisholt K.E., Haedersdal M., Togsverd-Bo K. (2019). Efficacy and safety of daylight photodynamic therapy after tailored pretreatment with ablative fractional laser or microdermabrasion: A randomized, side-by-side, single-blind trial in patients with actinic keratosis and large-area field cancerization. Br. J. Dermatol..

[41] Torezan L.A., Festa-Neto C. (2013). Cutaneous field cancerization: Clinical, histopathological and therapeutic aspects. An. Bras. Dermatol..

[42] See J.A., Shumack S., Murrell D.F., Rubel D.M., Fernández-Peñas P., Salmon R., Hewitt D., Foley P., Spelman L. (2016). Consensus recommendations on the use of daylight photodynamic therapy with methyl aminolevulinate cream for actinic keratoses in Australia. Australas. J. Dermatol..

[43] Braakhuis B.J., Tabor M.P., Kummer J.A., Leemans C.R., Brakenhoff R.H. (2003). A genetic explanation of Slaughter's concept of field cancerization: Evidence and clinical implications. Cancer Res..

[44] Goldenberg G., Perl M. (2014). Actinic keratosis: Update on field therapy. J. Clin. Aesthetic Dermatol..

[45] Schmitz L., Kahl P., Majores M., Bierhoff E., Stockfleth E., Dirschka T. (2016). Actinic keratosis: Correlation between clinical and histological classification systems. J. Eur. Acad. Dermatol. Venereol..

[46] Fitzmaurice S., Eisen D.B. (2016). Daylight Photodynamic Therapy: What is Known and What is Yet to be Determined. Dermatol. Surg..

[47] Wiegell S.R., Fabricius S., Gniadecka M., Stender I.M., Berne B., Kroon S., Andersen B.L., Mørk C., Sandberg C., Ibler K.S. (2012). Daylight-mediated photodynamic therapy of moderate to thick actinic keratoses of the face and scalp: A randomized multicentre study. Br. J. Dermatol..

[48] Pérez-Pérez L., García-Gavín J., Gilaberte Y. (2014). Daylight-mediated photodynamic therapy in Spain: Advantages and disadvantages. Actas Dermosifiliogr..

[49] Fai D., Romano I., Fai C., Cassano N., Vena G.A. (2016). Daylight photodynamic therapy with methyl aminolaevulinate in patients with actinic keratoses: A preliminary experience in Southern Italy. G. Ital. Dermatol. Venereol..

[50] Sotiriou E., Evangelou G., Papadavid E., Apalla Z., Vrani F., Vakirlis E., Panagiotou M., Stefanidou M., Pombou T., Krasagakis K. (2018). Conventional vs. daylight photodynamic therapy for patients with actinic keratosis on face and scalp: 12-month follow-up results of a randomized, intra-individual comparative analysis. J. Eur. Acad. Dermatol. Venereol..

[51] Cordey H., Valentine R., Lesar A., Moseley H., Eadie E., Ibbotson S. (2017). Daylight photodynamic therapy in Scotland. Scott. Med. J..

[52] Nissen C.V., Heerfordt I.M., Wiegell S.R., Mikkelsen C.S., Wulf H.C. (2017). Pretreatment with 5-Fluorouracil Cream Enhances the Efficacy of Daylight-mediated Photodynamic Therapy for Actinic Keratosis. Acta Derm. Venereol..

[53] Grinblat B., Galimberti G., Chouela E., Sanclemente G., Lopez M., Alcala D., Torezan L., Pantoja G. (2016). Daylight-mediated photodynamic therapy for actinic damage in Latin America: Consensus recommendations. Photodermatol. Photoimmunol. Photomed..

[54] Wulf H.C. (2016). Photodynamic Therapy in Daylight for Actinic Keratoses. JAMA Dermatol..

[55] Torezan L., Chaves Y., Niwa A., Sanches J.A., Festa-Neto C., Szeimies R.M. (2013). A pilot split-face study comparing conventional methyl aminolevulinate-photodynamic therapy (PDT) with microneedling-assisted PDT on actinically damaged skin. Dermatol. Surg..

[56] Heerfordt I.M., Wulf H.C. (2019). Daylight photodynamic therapy of actinic keratosis without curettage is as effective as with curettage: A randomized clinical trial. J. Eur. Acad. Dermatol. Venereol..

[57] Galimberti G.N. (2018). Calcipotriol as pretreatment prior to daylight-mediated photodynamic therapy in patients with actinic keratosis: A case series. Photodiagnosis Photodyn. Ther..

[58] Neittaanmäki-Perttu N., Karppinen T.T., Grönroos M., Tani T.T., Snellman E. (2014). Daylight photodynamic therapy for actinic keratoses: A randomized double-blinded nonsponsored prospective study comparing 5-aminolaevulinic acid nanoemulsion (BF-200) with methyl-5-aminolaevulinate. Br. J. Dermatol..

[59] Gutiérrez García-Rodrigo C., Pellegrini C., Piccioni A., Tambone S., Fargnoli M.C. (2019). Single versus two-treatment schedule of methyl aminolevulinate daylight photodynamic therapy for actinic keratosis of the face and scalp: An intra-patient randomized trial. Photodiagnosis Photodyn. Ther..

[60] Bernad I., Aguado L., Núñez-Córdoba J.M., Redondo P. (2020). Daylight photodynamic therapy for prevention of new actinic keratosis and keratinocyte carcinomas in organ transplants. A cryotherapy-controlled randomized clinical trial. J. Eur. Acad. Dermatol. Venereol..

[61] Levi A., Hodak E., Enk C.D., Snast I., Slodownik D., Lapidoth M. (2019). Daylight photodynamic therapy for the treatment of actinic cheilitis. Photodermatol. Photoimmunol. Photomed..

[62] Yazdani Abyaneh M.A., Falto-Aizpurua L., Griffith R.D., Nouri K. (2015). Photodynamic therapy for actinic cheilitis: A systematic review. Dermatol. Surg..

[63] Chaves Y.N., Torezan L.A., Lourenço S.V., Neto C.F. (2017). Evaluation of the efficacy of photodynamic therapy for the treatment of actinic cheilitis. Photodermatol. Photoimmunol. Photomed..

[64] Fernández-Guarino M., Mavura D., Fernández-González P., Chapa P., Ravazzano C., Jaén L., Rios L., Jaén P., Grossman H. (2020). Daylight photodynamic therapy is an option for the treatment of actinic keratosis in patients with xeroderma pigmentosum in Africa. Photodiagnosis Photodyn. Ther..

[65] Zamarrón A., García M., Río M.D., Larcher F., Juarranz Á. (2017). Effects of photodynamic therapy on dermal fibroblasts from xeroderma pigmentosum and Gorlin-Goltz syndrome patients. Oncotarget.

[66] Morton C., Szeimies R.M., Sidoroff A., Wennberg A.M., Basset-Seguin N., Calzavara-Pinton P., Gilaberte Y., Hofbauer G., Hunger R., Karrer S. (2015). European Dermatology Forum Guidelines on topical photodynamic therapy. Eur. J. Dermatol..

[67] Safar R., Alkhars A., Tallegas M., Korsaga-Some N., Machet L. (2019). Successful Treatment for Extensive Bowen’s Disease using Daylight-mediated Photodynamic Therapy. Acta Derm. Venereol..

[68] Martins C.C., Bakos R.M., Martins Costa M. (2020). Daylight photodynamic therapy for Bowen’s disease. An. Bras. Dermatol..

[69] Wiegell S.R., Skødt V., Wulf H.C. (2014). Daylight-mediated photodynamic therapy of basal cell carcinomas—An explorative study. J. Eur. Acad. Dermatol. Venereol..

[70] Paolino G., Didona D., Scarnò M., Tallarico M., Cantoresi F., Calvieri S., Mercuri S.R., Piccolo D., Bottoni U., Kyriakou A. (2019). Sequential treatment of daylight photodynamic therapy and imiquimod 5% cream for the treatment of superficial basal cell carcinoma on sun exposed areas. Dermatol. Ther..

[71] Zhang L., Zhang Y., Liu X., Shi L., Wang P., Zhang H., Zhou Z., Zhao Y., Zhang G., Wang X. (2020). Conventional versus daylight photodynamic therapy for acne vulgaris: A randomized and prospective clinical study in China. Photodiagnosis Photodyn. Ther..

[72] Le Pillouer-Prost A., Cartier H. (2016). Photodynamic Photorejuvenation: A Review. Dermatol. Surg..

[73] Philipp-Dormston W.G., Sanclemente G., Torezan L., Tretti Clementoni M., Le Pillouer-Prost A., Cartier H., Szeimies R.M., Bjerring P. (2016). Daylight photodynamic therapy with MAL cream for large-scale photodamaged skin based on the concept of ‘actinic field damage’: Recommendations of an international expert group. J. Eur. Acad.Dermatol. Venereol..

